# Efficient Capture and Raman Analysis of Circulating Tumor Cells by Nano-Undulated AgNPs-rGO Composite SERS Substrates

**DOI:** 10.3390/s20185089

**Published:** 2020-09-07

**Authors:** Jong-Eun Park, Nuri Oh, Hyeono Nam, Ji-Ho Park, Sanha Kim, Jessie S. Jeon, Minyang Yang

**Affiliations:** 1 Department of Mechanical Engineering, Korea Advanced Institute of Science and Technology, Daejeon 34141, Korea; pje7031@kaist.ac.kr (J.-E.P.); namtree@kaist.ac.kr (H.N.); sanhkim@kaist.ac.kr (S.K.); 2 Department of Bio and Brain Engineering, Korea Advanced Institute of Science and Technology, Daejeon 34141, Korea; ohnuri@kaist.ac.kr (N.O.); jihopark@kaist.ac.kr (J.-H.P.); 3 Center for Systems Biology, Massachusetts General Hospital, Boston, MA 02114, USA; 4 Department of Mechanical Engineering, State University of New York Korea, Incheon 21985, Korea

**Keywords:** circulating tumor cells, silver nanoparticles-reduced graphene oxide composites, nano-undulated surface, electric field-assisted laser reduction, efficient capture, surface-enhanced Raman scattering spectroscopy

## Abstract

The analysis of circulating tumor cells (CTCs) in the peripheral blood of cancer patients is critical in clinical research for further investigation of tumor progression and metastasis. In this study, we present a novel surface-enhanced Raman scattering (SERS) substrate for the efficient capture and characterization of cancer cells using silver nanoparticles-reduced graphene oxide (AgNPs-rGO) composites. A pulsed laser reduction of silver nanowire-graphene oxide (AgNW-GO) mixture films induces hot-spot formations among AgNPs and artificial biointerfaces consisting of rGOs. We also use in situ electric field-assisted fabrication methods to enhance the roughness of the SERS substrate. The AgNW-GO mixture films, well suited for the proposed process due to its inherent electrophoretic motion, is adjusted between indium tin oxide (ITO) transparent electrodes and the nano-undulated surface is generated by applying direct-current (DC) electric fields during the laser process. As a result, MCF7 breast cancer cells are efficiently captured on the AgNPs-rGO substrates, about four times higher than the AgNWs-GO films, and the captured living cells are successfully analyzed by SERS spectroscopy. Our newly designed bifunctional substrate can be applied as an effective system for the capture and characterization of CTCs.

## 1. Introduction

Circulating tumor cells (CTCs) shedding from primary or metastatic lesions and entering into bloodstream are intimately related with the hematogenous spread of cancer to distant organs, resulting in most cancer deaths. Thus, CTCs are known as an important cancer biomarker and they also provide a predictive role in detection of cancer recurrence, prognosis and treatment procedures for the metastatic cancer patient [1,2]. Despite the huge interests in clinics, capturing and detecting CTCs directly from human blood is still a big challenge. This is because CTCs are extremely rare in the blood stream; less than few hundred among a billion hematologic cells in 1.0 mL of blood [3]. Therefore, continuous efforts are being made to enhance technologies that can capture CTCs with high efficiency followed by detection with high sensitivity and specificity [4].

To isolate and capture CTCs from peripheral blood, a number of strategies including cell-size membrane filtration, immunomagnetic bead separation, antibody-based purification and microfluidic chips have been developed in which microfabrication technologies are utilized as enrichment methods [5]. Although these approaches advanced the CTC capture performance, they failed to achieve high efficiency, purity, viability and throughput simultaneously. For example, isolation methods relying on size differences cause impurity as CTCs are of a similar size to leucocytes. In addition, they suffer clogging in the membrane structure. Immunomagnetic separation and antibody-based purification approaches are limited by the low capture efficiency and heterogeneous expression of CTCs occurred from epithelial-mesenchymal transition (EMT) [1,2,3,4]. Microfluidic devices can increase the capture efficiency by adapting high-aspect-ratio microposts, herringbone, sinusoidal and staggered obstacle channels [6]. However, fabricating complex structures is expensive and time-consuming and the operation principles are also complicated, which hinders their wide usage toward high-throughput and downstream molecular assays of CTCs [7].

Recently, nanomaterials such as magnetic nanoparticles [8], nanopillars [9,10], nanowires [11], carbon nanotubes [12], graphene oxide [13] and nanoroughened surfaces have been proposed to overcome the limits of previous research by utilizing their intrinsic characteristics [14]. The high surface-area-to-volume ratio and facile functionalization are beneficial for mimicking the natural cell microenvironment [15,16]. Furthermore, the physical and chemical properties of interaction between cells and the extracellular matrix can be applicable to cell-capture platforms for the improvement of capture yield, purity and throughput through nanomaterials [17]. In particular, graphene based materials have been regarded as one of the promising materials as they can effectively serve as biointerfaces for water insoluble cancer drug carriers, photothermal cancer therapy and bacteria or DNA biosensors with biocompatible properties [18,19,20,21].

In the case of detection and analysis of CTCs, fluorescence technology has been the most conventionally used method. The technique can selectively target the cell components by imaging with labeling fluorophores or quantum dots (QDs) [22]. However, fluorescence imaging suffers from photobleaching, broad absorption and emission band spectra of the dyes as well as a strong auto-fluorescence background in the cell and tissue microenvironment. Thus, in fluorescence microscopy, it is hard to detect a small amount of protein due to its weak fluorescence signal [23,24]. These characteristics have been prominently featured in rare CTC detection. Furthermore, a cell releasing process that can reduce the capture yields and viability of CTCs is required to perform molecular diagnostics after the CTC isolation [25]. On the other hand, the surface-enhanced Raman scattering (SERS) technology offers even a single molecule detection sensitivity owing to the significant electromagnetic field enhancement from the localized surface plasmon resonance (LSPR) on nearby rough metallic surfaces. The narrow fingerprint-like spectral lines of the SERS signal provides high specificity of the analytes, which can be easily distinguished from other proteins and cells in biological samples. Therefore, the SERS technology gives a new opportunity in the detection and analysis of CTCs by overwhelming the sensitivity and specificity of fluorescence-based methods [26,27]. Nima et al. developed silver-coated gold nanorods for the highly specific multiplexing targeting and multicolor identification of tumor cells in human peripheral blood [28]. In another study, Zhang et al. demonstrated capturing and SERS imaging of CTCs in whole human blood based on a nitrocellulose substrate and gold nanoparticles [29]. Although these studies enabled the capturing of tumor cells, there are limitations that these require the additional labeling of SERS probes or tags, which are metal nanoparticles conjugated with Raman-active molecules and binding ligands, such as antibodies, to target specific cell surfaces or proteins after CTC enrichment. Furthermore, the loss of CTCs can be induced during the labelling process or in the post releasing and molecular diagnostics process.

**Figure 1:**
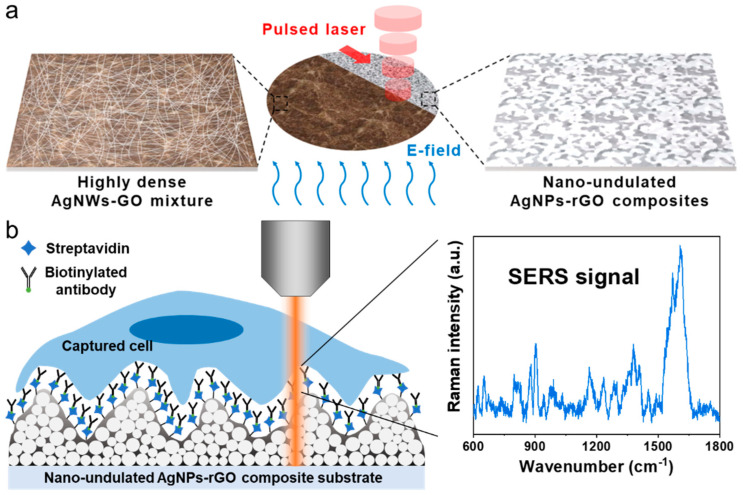
Schematic illustration. (**a**) The electric field-assisted pulsed laser reduction for the fabrication of the nano-undulated AgNPs-rGO composite substrate; (**b**) Capture and surface-enhanced Raman scattering (SERS) analysis of circulating tumor cells (CTCs). The highly soft nanostructure surface functionalized with an anti-EpCAM antibody improved the capture efficiency. Hot spots generated from the narrow nanogaps in the substrates greatly increased the SERS signals of the captured cells.

Many research groups have developed various artificial biointerfaces such as polystyrene [10], polypyrrole [30], poly(3,4-ethylenedioxy)thiophenes [31] and graphene oxides for the efficient capture and detection of tumor-derived biomarkers [16]. In spite of their excellent capture efficiency and limit of detection (LOD), complex post processes, including cell release, for investigating genetic information of tumor cells hinder its clinical application due to the low cell viability. In order to enable molecular characterization and cell analysis without post processes, SERS spectroscopy employing nanogap-rich metal nanoparticles-based structures can be an effective analytic tool [32,33]. Although SERS spectroscopy can investigate CTCs directly, few researches have reported the capture efficiency at the same time. Herein, we suggest silver nanoparticles-reduced graphene oxide (AgNPs-rGO) composites for the highly efficient capture and Raman analysis of rare CTCs. We fabricated the new functional surface via an in situ electric field-assisted laser reduction process, which was a cost-effective method, as shown in Figure 1a. The antibody-modified rGO films took the roll of biointerfaces for efficient CTC capture while nanogaps between the AgNPs generated the hot spots to enhance the Raman signal of the captured molecules. As a label-free analyzing concept, instead of using molecular diagnostics, we characterized the captured living cells on the AgNPs-rGO substrate by SERS spectroscopy, which did not require a cell releasing process. After incubation of the cells, characteristic Raman bands of biomolecules on the captured sites were substantially enhanced so that the captured cancer cells could be differentiated from the normal cells through SERS signals, as illustrated in Figure 1b. To discriminate between the cancer and normal cells through the SERS spectra quantitatively, principal component analysis (PCA) was applied, which spanned the dataset onto orthogonal vector spaces that maximized the projected data variance in the space by the linear combination of the variables. This orthogonal linear transformation dimensionally reduced the dataset through the significant principal components selection and was appropriate to classify the Raman spectra of the cells [34]. Using the newly fabricated AgNPs-rGO composite surfaces, we demonstrated the efficient capturing of MCF7 breast malignant cells and distinguished them from MCF10A breast non-malignant cells without complex and time-consuming post processes.

## 2. Materials and Methods

### 2.1. Materials

A dispersion solution of 0.15 wt% AgNWs was purchased from Nanophyxis. The GO solution was prepared by modifying Hummer’s method [18,35,36,37]. A nylon filter with 200 nm pore size was obtained from Whatman. HPLC grade deionized (DI) water and methanol was procured from Burdick and Jackson. Phosphate-buffered saline (PBS) was supplied by Welgene. Pyrene carboxylic acid (PrCA), N-hydroxysulfosuccinimide (Sulfo-NHS) and N-(3-dimethylaminopropyl)-N-ethylcarbodiimide hydrochloride (EDC), Triton X-100, 4′6-Diamidino-2-phenylindol (DAPI) and rhodamine 6G (R6G) were purchased from Sigma Aldrich. Straptavidin (SA) and a 4-well Lab-Tek chamber slide for cell culture plates were obtained from Thermo Fisher Scientific. Biotinylated anti-human epithelial cell adhesion molecules (EpCAM)TROP1 (biotinylated anti-EpCAM) and human (EpCAM)TROP1 Alexa Flour 488-conjugated antibodies were procured from R&D Systems. The MCF7 and MCF10A breast cancer and non-cancer cell lines were supplied by ATCC. Dulbecco’s modified Eagle’s medium (DMEM) and mammary epithelial cell growth medium (MEGM) were purchased from Lonza. Fetal bovine serum (FBS) antibiotic-antimycotic (100×) solution were obtained from Gibco. Cholera toxin was procured from List Biological Laboratories.

### 2.2. Preparation of AgNWs-GO Mixture Composites

Different amounts (3.0, 1.0, 0.6 and 0.4 mL) of GO solution (4 mg/mL) were added to 10 mL of AgNWs dispersion solution to form the various weight ratio of AgNWs-GO composites. These are referred to as AgNWs-GO1 (7:1), AgNWs-GO2 (5:1), AgNWs-GO3 (3:1) and AgNWs-GO4 (1:1) throughout this study. Sonicated AgNWs-GO mixture dispersions were vacuum filtrated onto a nylon membrane and transferred onto a glass substrate to obtain the AgNWs-GO composite films.

### 2.3. Pulsed Laser Reduction of AgNWs-GO Films

A 1064 nm wavelength pulsed Yitterbium laser (IPG Photonics) was used to transform the AgNWs-GO composite films under ambient conditions. A collimated laser beam with a pulse width of 200 ns, pulse repetition rate of 20 kHz and average power of 8 W was delivered by a galvanometer scanner (Hurryscan, Scanlab) through an f-theta lens (Linos) with 700 mm focal length. The focused laser beam with 70 μm diameter was irradiated onto the AgNWs-GO films and raster scanned with a hatch size of 35 μm and scanning speed of 500 mm/s. The entire AgNWs-GO mixture composites were then photothermally reduced onto AgNPs-rGO composite films within 10 s.

### 2.4. Fabrication of Nano-Undulated AgNPs-rGO Composite SERS Substrates

In order to roughen the surface of the AgNPs-rGO composites, an in situ electric field-assisted fabrication method was utilized. The AgNWs-GO composite films were horizontally placed in the midst of two parallel ITO substrates. The distance between them was 3 mm and the upper and lower electrodes were anode and cathode, respectively, before the laser treatment. When applying DC voltages of 7.5, 15, 22.5 and 30 V, and holding the potentials during the laser process, the nano-undulated surface was formed due to the electrophoretic motion of the AgNPs and rGOs.

### 2.5. Characterization of AgNPs-rGO Substrates

Images of scanning electron microscopy (SEM, Nova230, FEI) and transmission electron microscopy (TEM, Talos F200X, FEI) with an energy dispersive X-ray spectroscopy (EDS) detector were observed to investigate the surface morphology and chemical composition of the substrates. ImageJ software was used for the calculation of the particle size distribution of the substrate. Raman spectra of the AgNWs-GO composite films were measured using a dispersive Raman spectrometer (LabRAM Aramis, Horiba Jobin Yvon) equipped with an excitation wavelength of 514 nm He-Ne and 633 nm Ar laser. The laser beam of 1.25 mW and 5 mW power was focused on the substrate with 1.24 μm (514 nm) and 1.52 μm (633 nm) spot sizes through a 50×/0.5NA objective lens, 1 s exposure time and backscattered with 1200 lines/mm grating. An atomic force microscopy (AFM) system (NX10, Park systems) was used to measure the roughness of the AgNPs-rGO substrates. The contact angles and stiffness of the AgNWs and AgNWs-GO films were measured by a contact angle analyzer (Phoenix 300 Plus, SEO) and a nanoindentation test system (iNano Nanoindenter, KLA-Tencor), respectively.

To investigate the SERS performance, 1 μL of aqueous R6G solutions in the range of 10^−4^–10^−8^ M were dropped on the fabricated substrates and dried in the air. Raman spectra of the R6G were observed using the same Raman spectrometer with a 633 nm wavelength, 1.25 mW power, 50× magnification and 5 s exposure time. We calibrated the all Raman spectra at 521 cm^−1^ of a bare silicon wafer before each measurement. A total of 10^−6^ M R6G Raman spectra of 50 random spots, which were 10 points per each sample and a total of five SERS substrates, were measured in the reproducibility test. Furthermore, to evaluate the stability of the SERS substrate, we measured the Raman spectra of 10^−6^ M R6G solutions every week for a month.

### 2.6. Surface Functionalization of the AgNPs-rGO Composite Substrates

The AgNPs-rGO substrates were soaked into 10 mL PrCA solution (3 mg/mL in methanol) for 24 h and dried with nitrogen gas. The PrCA-AgNPs-rGO substrates were modified with a 3 mL solution of Sulfo-NHS (0.01 M) and EDC (0.2 M) in DI water for 2 h; then, after washing with PBS three times, 3 mL SA (10 μg/mL in PBS) was added to the Sulfo-NHS/EDC modified PrCA-AgNPs-rGO substrates for 1 h. Next, after washing with PBS for three times, 25 μL of biotinylated anti-EpCAM and Alexa Flour 488-conjugated antibodies (10 μg/mL in PBS) were dropped onto the SA treated substrate and incubated at 37 °C for 1 h and rinsed with PBS for three times.

### 2.7. Cell Culture and Capture

MCF7 and MCF10A cells were cultured in a DMEM medium containing 10% (*v*/*v*) FBS and 1% (*v*/*v*) antibiotic-antimycotic solution and an MEGM medium containing 100 ng/mL cholera toxin and 1% (*v*/*v*) antibiotic-antimycotic solution, respectively, at 37 °C under 5% of CO_2_. After the anti-EpCAM functionalized substrates were placed into a size-matched 4-well cell culture plate, 1 mL of MCF7 cell suspensions (10^5^ cells per mL) were loaded onto the substrates and incubated for the predetermined capturing time in 37 °C and 5% of CO_2_ environment. These were then rinsed with PBS three times and the captured cells were fixed with 4 wt% PFA (in PBS) for 20 min and treated with 0.2 wt% Triton X-100 (in PBS) for 10 min to improve cellular permeability. This was followed by dyeing utilizing DAPI and a rhodamine-phalloidin solution (2 μg/mL in DI water and 165 nM in PBS, respectively). MCF10A cells were captured on the substrates without the surface functionalization. Fluorescence images of the cells were obtained by confocal laser scanning microscope (LSM 880, Carl Zeiss) and the capture efficiency was evaluated using ImageJ software.

### 2.8. SERS Measurements and Data Analysis

SERS spectra of the captured living cells on the substrate were measured by Raman mapping using the dispersive Raman spectrometer (LabRAM Aramis, Horiba Jobin Yvon) equipped with a 633 nm wavelength excitation Ar laser. The focused light with 1.52 μm beam diameter and 1.25 mW laser power was raster scanned onto the SERS substrate through 1.5 μm step size in the spectral range of 600–1800 cm^−1^ with 5 s integration time. Smoothing, background subtraction and normalization were then applied to the measured Raman spectra using the BEADS toolbox and built-in function of MATLAB software [38]. After that, principal component analysis (PCA) was performed on each spectrum with variables of 600–1800 cm^−1^ using MATLAB and the first and second principal components were selected for the analysis. Briefly, the dataset consisted of equally distributed 1401 variables from the wavenumbers of 600–1800 cm^−1^ involving the corresponding measured SERS spectra from the cells and were inserted into the input data followed by the calculation of the covariance matrix and its eigenvalues and eigenvectors. The first and second largest eigenvalues are the variances of the first and second principal components (PC1 and PC2), respectively, and each SERS spectrum could be linear transformed onto the PC1 and PC2 through the corresponded eigenvectors. The output data showed the projected data on the principal components resulting in the score values and the components of the eigenvectors represented loading plots of the principal components for the variables.

## 3. Results and Discussion

### 3.1. Composites as Substrates

**Figure 2:**
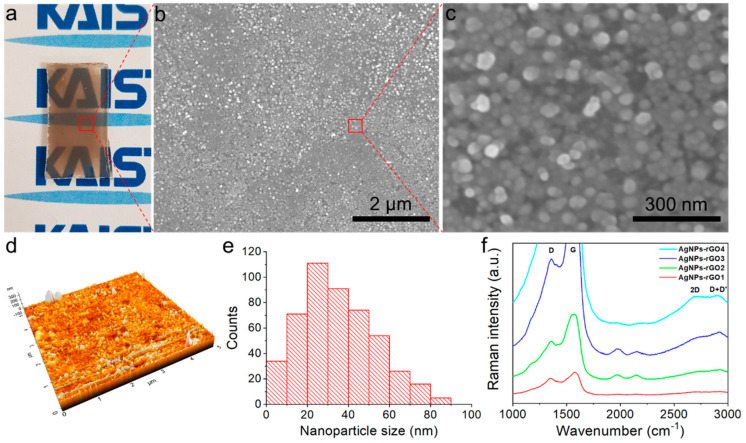
Characterization of the AgNPs-rGO composites. (**a**) Photograph of the AgNPs-rGO1 SERS substrate; (**b**,**c**) The SEM images clearly showed the narrow nanogap-rich structures; (**d**) Atomic force microscopy (AFM) images of the AgNPs-rGO1 substrate; (**e**) Particle size distribution of the AgNPs-rGO1 substrate exhibiting the median size of AgNPs was 33 nm; (**f**) Raman spectra of various AgNPs-rGO composites. The D and G Raman bands of the AgNPs-rGO3 and AgNPs-rGO4 substrates were not figured as the signals were saturated.

We first deposited a highly dense film of AgNWs and GO mixture onto a flat substrate through vacuum filtration. We then used a 1064 nm pulsed laser beam for the in situ electric field-assisted laser reduction process. The laser reduction involved both the photothermal reduction of GO and the morphological dimension change from AgNW (1-D) to AgNP (0-D) via irradiation of the focused pulse laser beam. Figure 2a shows a photograph of the AgNPs-rGO composite surface after the laser exposure. We formed a series of AgNWs-GO mixture films with different weight ratios onto glass substrates via vacuum filtration and a transfer process. We fabricated AgNWs-GO mixture films having different weight ratios of the AgNWs and the GO flakes as 7:1, 5:1, 3:1 and 1:1, mentioned as the AgNWs-GO1, AgNWs-GO2, AgNWs-GO3 and AgNWs-GO4, respectively. After the photothermal laser reduction, each of the AgNWs-GO films were reduced to the AgNPs-rGO1, AgNPs-rGO2, AgNPs-rGO3 and AgNPs-rGO4 composites, respectively.

Figure 2b,c show SEM images and Figure 2d shows an AFM image of the AgNPs-rGO1 substrate consisting of the AgNPs and rGO derivatives. We observed narrow nanogap-rich structures in the high magnification SEM images and roughness of 19 ± 3 nm, as depicted in Table 1. In Figure 2e, the size distribution of the AgNPs showed a median nanoparticle size of 33 nm in the unit area (1 μm × 1 μm) utilizing ImageJ software. This nanostructure based on the AgNPs, which had a few tens of nm sized diameter and roughness with a sub-10 nm nanogap, is well suited for the high enhancement of the Raman signal [37,39,40]. The strong electromagnetic fields produced by the localized surface plasmon resonances at the surface of the AgNPs were a major mechanism of SERS, which showed that electromagnetic mechanism (EM) and hot spots generated from the narrow nanogaps near the AgNPs could be expected to greatly increase the SERS signals of the cells. In Figure S1, a high-angle annular dark-field (HAADF) image of the AgNPs-rGO composites and the corresponding elemental maps for Ag and C also show the nanogaps conformably covered with the rGO nanosheet. For the investigation of the AgNPs-rGO composites in terms of the rGO nanosheet, we obtained the Raman spectra of the various AgNPs-rGO substrates with the different GO weight ratios in the AgNWs-GO films using an input laser power of 5 mW. Typical Raman spectra of D (1354 cm^−1^), G (1580 cm^−1^), 2D (2690 cm^−1^) and D+D’ (2903 cm^−1^) bands in rGO sheets are illustrated in Figure 2f [35,36,41]. The 2D peak of the rGO represented the graphene formulation induced by the photothermal laser reduction. The overall increase of the Raman intensities with the increase of the GO weight ratio indicated that the substrates contained a higher rGO content [42]. Furthermore, the intensity ratio of the 2D peak to the G peak (I_2D_/I_G_) was decreased as the GO weight ratio increased, as shown in Figure 2f. This implied that there was more GO content in the AgNWs-GO films when there were more graphene layers in the AgNPs-rGO composites [36,40,43]. However, in regard to the performance of the SERS substrates, it is noteworthy that multi-layers of rGO sheets resulting from a high GO concentration could reduce the Raman signals of analytes [33,37]. In order to examine the possibility that our composite SERS substrates interrupted the Raman signals of analytes, we measured the Raman spectra of AgNPs-rGO1 substrates using a low laser power of 1.25 mW, as shown in Figure S2. Raw data were represented to verify as a role of background signals and the Raman spectra were significantly reduced when compared with the case of 5 mW laser power at both 514 and 633 nm excitation wavelength. An absence of distinct Raman peaks of rGO, including the D and G bands and any other Raman signals, demonstrates the superiority of the substrate in terms of the background signals in Raman spectra.

We investigated the SERS effect of the AgNPs-rGO1 substrates using R6G solutions as shown in Figure S3. The strong characteristic peak of R6G at 612 cm^−1^ corresponding to a C−C ring in-plane bending mode was observed from 10^−4^ M to 10^−8^ M. The limit of detection (LOD) was calculated from the average intensity of the blank substrate plus three times the standard deviation at 612 cm^−1^ using the logarithmic graph and the least-square fit line; the LOD was then 2.0 nM [44]. We compared the LOD of R6G with other methods in Table S1. The enhancement factor (EF) was calculated according to the following equation [45]:

EF = (*I*_SERS_/*I*_bulk_) × (*N*_bulk_/*N*_SERS_) (1)

where *I*_SERS_ and *I*_bulk_ represent the intensities of SERS and normal Raman scattering, respectively. *N*_bulk_ and *N*_SERS_ are the numbers of molecules in the volume of contributing normal Raman scattering and SERS, respectively, as detailed in Supplementary Materials. The calculated EF was 4.88 × 10^5^ and distinctive molecular identification of the cell components could be expected [32]. To evaluate the reproducibility of the substrates, the relative standard deviation (RSD) values at 612, 773, 1362 and 1650 cm^−1^ were estimated from the 50 collected Raman spectra of R6G. In Figure S4, the calculated RSD showed less than 20%, which is appropriate for the practical use. In addition, the stability test exhibited the SERS intensity, which is maintained without the signal decreasing compared with the freshly fabricated substrate for a month, as shown in Figure S5. It might seem to be due to the protection of the GO nanosheets and this is suitable for long time usage.

### 3.2. Application of In Situ Electric Field for Surface Roughness Enhancement

**Figure 3:**
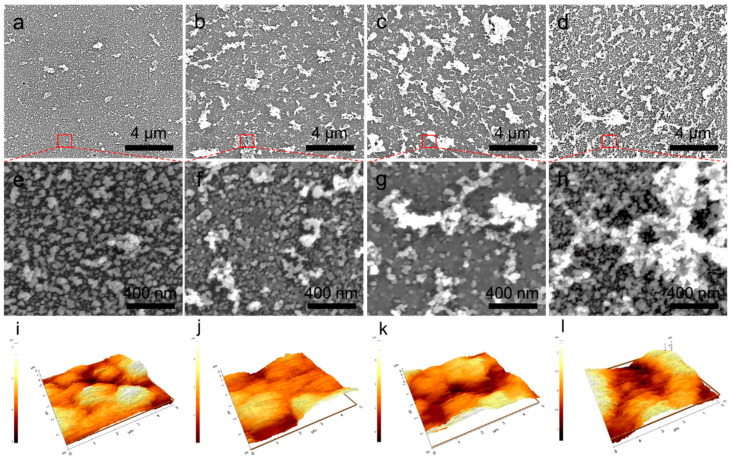
Characterization of the AgNPs-rGO composites fabricated at the different amplitudes of DC electric fields during the laser reduction. (**a**–**d**) SEM images of the AgNPs-rGO1-E1, the AgNPs-rGO1-E2, the AgNPs-rGO1-E3 and the AgNPs-rGO1-E4 substrates applied with 7.5 V, 15 V, 22.5 V and 30 V, respectively; (**e**–**h**) The high magnification SEM images; (**i**–**l**) AFM results of each of the AgNPs-rGO substrates.

We employed in situ electric fields during the laser treatment of the AgNWs-GO mixture films to enhance the roughness and increase the capture efficiency. During the laser reduction with direct current (DC) electric fields, the negatively charged AgNPs-rGO composite fragments experienced gas-phase electrophoretic motion, which formulated the nano-undulated surface. The electrophoretic motion of the inherently negative charged AgNPs and rGO hybrid flakes and electrically conducting ion plume formation in the laser reduction process could facilitate the construction of the nano-undulated surface from this simple and controllable process. Figure 3a–h show SEM images of the AgNPs-rGO composite substrates after the fabrication with different conditions of the applied DC electric fields, which are referred to as AgNPs-rGO1-E1, AgNPs-rGO1-E2, AgNPs-rGO1-E3 and AgNPs-rGO1-E4, respectively. We observed agglomeration of the AgNPs and rGO flakes forming the nano-undulated surfaces, which had a larger size with the increase of the applied electric fields. Root-mean-squire (RMS) roughness of the nano-undulated AgNPs-rGO composites were also measured by utilizing AFM according to applying the various electric fields, as shown in Figure 3i–l and Table 1. When the applied DC electric fields increased during the laser reduction process, the surface roughness of the AgNPs-rGO substrates also increased up to around 700 nm, in the case of the AgNPs-rGO1-E3 substrate. The increase in the roughness is attributed to the gas-phase electrophoretic motion of the negatively charged AgNPs-rGO composite fragments and the Ag ions. Immediately after irradiation of the nanosecond pulsed laser beam onto the AgNWs-GO films, the melted and resolidified AgNWs and the photothermally reduced GOs merged and formulated the AgNPs-rGO composite fragments and the ionized AgNWs generated a plasma plume above the substrate. In this procedure, the numerous oxygen-containing functional groups of the graphene derivatives contributed to the net negative charge of the AgNPs-rGO composite fragments undergoing the electrophoretic motion to the upper anode in the applied DC electric fields whereby they formulated the nano-undulated surface [46,47,48,49]. As the electric field increased, the nano-undulated surfaces were enhanced by the larger electrophoretic force that brought about an intense merging motion of the AgNPs and rGO flakes, which formed the large agglomeration on the substrate as depicted in Figure 3a–h [50].

Meanwhile, the plasma plume induced by the laser ablation radially propagated over several centimeters in a few microseconds of temporally delayed time [51,52,53]. In this study, the ITO electrodes were placed in the plasma region and adjusted in parallel with both sides of the substrates. The ion plume then acted as an electrically conducting medium during the laser reduction process and the Ag ions were forced to be at a location down to the cathode through the DC electric fields. This may have facilitated better formation of the nano-undulated surface corresponding to the increase of the electric fields. It was also noteworthy that the roughness of the AgNPs-rGO1-E4 substrate, applied with the highest potential, was lower than that of the AgNPs-rGO1-E3 substrates. This implies the limit of the roughness enhancement owes to the congealment and stabilization of the nanostructures as the surrounding temperature gradually decreases.

In terms of SERS enhancement, although the nano-undulations could induce a higher density of hot spots near the undulated surface with higher SERS enhancement [54], we did not utilize the nano-undulated surface for the SERS enhancement due to relatively high heterogeneity in the 3D structures [33], but the increase of capture efficiency through the nanotopographic sensing of the cells [10,14].

### 3.3. Capture Efficiency of Various AgNPs-rGO Composites Substrates

We selected MCF7 cells, which are EpCAM positive breast cancer cell lines, to examine the cell capturing performance of the nano-undulated AgNPs-rGO composites for CTC analysis. The pyrene carboxylic acid (PrCA) was introduced to form abundant carboxyl groups onto the substrate by π-π stacking, thereby conjugating anti-EpCAMs onto the substrate [16,55]. We used the chemical abbreviation PrCA to avoid redundancy with the acronym PCA. Streptavidin (SA) was then immobilized onto the surface of the PrCA-AgNPs-rGO substrate by assistance of N-hydroxysulfosuccinimide (Sulfo-NHS) and N-(3-dimethylaminopropyl)-N-ethylcarbodiimide hydrochloride (EDC) chemistry. Finally, a biotinylated anti epithelial cell adhesion molecule (anti-EpCAM) antibody was conjugated onto the SA-AgNPs-rGO substrate by avidin-biotin interactions [9,10,56]. An Alexa Flour 488-conjugated antibody was utilized to inspect the uniform distribution on the surface, as shown in Figure S6. Following this, 1 mL MCF7 cell suspensions (1 × 10^5^ cells per mL) were loaded and captured onto the anti-EpCAM modified AgNPs-rGO substrates. The fluorescence microscopic images of MCF7 cells captured and followed by staining with DAPI and rhodamine-phalloidin on the various substrates are illustrated in Figure S7.

**Figure 4:**
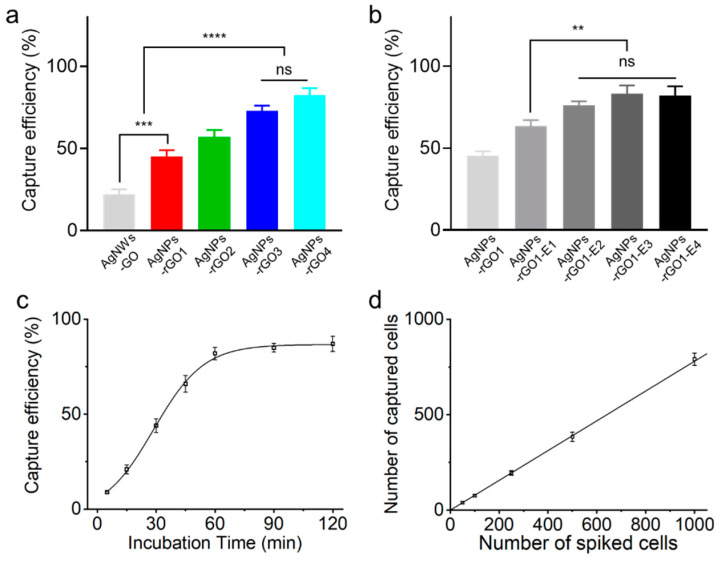
Capture efficiency of MCF7 cancer cells on the various AgNPs-rGO composite substrates with different GO weight ratios. (**a**) Fabricated with different applied electric fields; (**b**) Capture efficiency of MCF7 cancer cells on the AgNPs-rGO1-E4 substrate at different incubation times and (**c**) according to different cell concentrations; (**d**) Data representing the mean ± standard deviation (n = 3, ** *p* < 0.05, *** *p* < 0.01 and **** *p* < 0.001; one-way analysis followed by Tukey’s test).

Figure 4a shows the capture efficiency of the MCF7 cells on the AgNPs-rGO composite substrates with different GO to AgNW weight ratios. The capture efficiency of the MCF7 cells from the composite substrates increased with higher GO weight ratios. After 1 h incubation time, the capture efficiency of MCF7 cells on the AgNPs-rGO3 and the AgNPs-rGO4 substrates reached up to 73 ± 3.2% and 82 ± 4.4%, respectively, which were not statistically significant in the one-way analysis of variance followed by Tukey’s test. In contrast, the AgNWs-GO substrate, which was not laser treated and used as a control substrate, exhibited only 22 ± 3.3% and was statistically significant to the AgNPs-rGO1 substrate of 45 ± 4.0% with a *p*-value of 0.001. The group of AgNWs-GO and AgNPs-rGO1 and the group of AgNPs-rGO3 and AgNPs-rGO4 were also statistically significant with a *p*-value of 0.0001. The results indicated that the rGO flakes played an important role as an artificial biointerface in capturing the cells. The rGOs in the composites provided functional groups for antibody modification of the surface and reduced the stiffness of the AgNPs-rGO composites for mimicking the soft extracellular matrix. We measured the contact angles and Young’s modulus of AgNWs and AgNWs-GO1 films to investigate hydrophilic modification and the stiffness of the composites, as shown in Figure S8. The AgNWs-GO1 films resulted in the reduced contact angle and slightly increased stiffness of about 2 GPa but exhibited similar stiffness with the polystyrene nanotubes, which showed the effective cell capturing [10]. These surfaces induced adhesion preference of tumor cells to the 3-D nanostructured substrate. It is possible that the high concentration ratio of the rGO led the substrate to be softer and enhanced the cell-matrix interactions [10,16,31]. However, as shown in Figure 2f, because the high concentrations and multi layers of rGO could interrupt the SERS analysis of CTCs due to the Raman bands of rGOs [33,36], we selected the AgNPs-rGO1 substrate for the further study.

The increased roughness of the AgNPs-rGO composites by using the electric field-assisted fabrication could reduce the level of rGO contents in the substrate but maintain the cell capturing capability. The capture efficiency of the MCF7 cells on the various AgNPs-rGO composites with different roughness is shown in Figure 4b. After 1 h incubation time, the capture efficiencies of MCF7 cells on the AgNPs-rGO1-E1 substrate exhibited 63 ± 3.6%; approximately a 20% increase compared with the AgNPs-rGO1 substrate. On the AgNPs-rGO1-E2, AgNPs-rGO1-E3 and AgNPs-rGO1-E4 substrates, the capture efficiencies reached up to 76 ± 2.3%, 83 ± 4.8% and 82 ± 5.7%, respectively, returning not statistically significant results for each other. The AgNPs-rGO1-E1 substrate and the group of AgNPs-rGO1-E2, AgNPs-rGO1-E3 and AgNPs-rGO1-E4 substrates were statistically significant with a *p*-value of 0.01. As the roughness of the substrate increased, known as the nanostructure matching effect, the nano-undulated surface topography was more interactive with cell components such as actin, cytoskeleton, vinculin and filopodia [14,16,31]. They enhanced adhesion strength between the cells and the substrates, contributing to the spread of the actin filaments onto the surface. The synergistic effect from the 3D nanostructured matching effect and the soft cell-matrix interactions induced by the rGO nanosheets likely resulted in much higher focal adhesions and capture efficiency of the tumor cells.

We further quantified the capture efficiency at various incubation times and spiked cell numbers using the AgNPs-rGO1-E4 substrate. Figure 4c shows the capture efficiency of MCF7 cells for different incubation times. The capture efficiency of MCF7 cells gradually increased in the first hour and reached up to almost 90% after two hours of incubation. We selected one hour as an optimal capturing condition, which shows the capture efficiency over 80% enough to Raman analysis. The capture efficiency of MCF7 cells at a different number of spiked cells under the optimal condition is shown in Figure 4d, resulting in the average of 39, 76, 195, 384 and 791 captured cells when using 50, 100, 250, 500 and 1000 cells spiked into 1 mL PBS, respectively. The result represented almost 80% of high capture efficiency at the low cell concentrations, applicable to the low number of CTCs in clinical samples.

### 3.4. SERS Measurement and Analysis of Captured Cancer Cells

**Figure 5:**
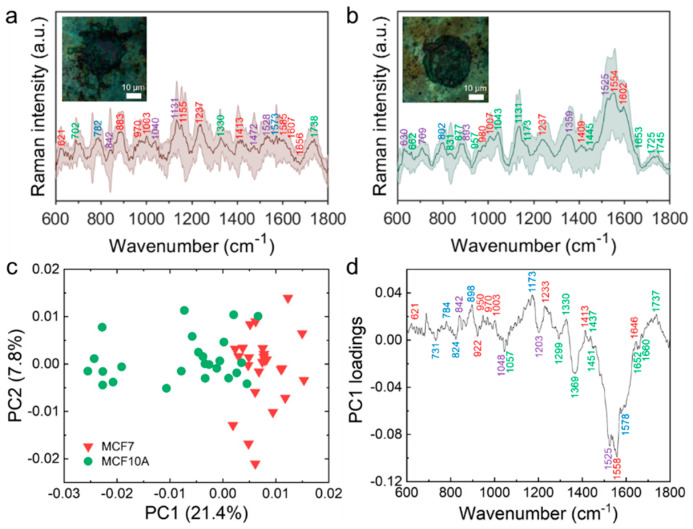
SERS measurement and PCA analysis of the captured living malignant MCF7 cells and non-malignant MCF10A cells. The SERS spectra of MCF7 (**a**) and MCF10A cells (**b**) The measured spectra were followed by Savitzky–Golay smoothing (width = 21, order = 5), background subtraction and normalization. The inset is OM images of the MCF7 and MCF10A cells, respectively. (**c**) Scatter plot of the score values of each Raman spectrum for PC1 and PC2 from the MCF7 (red triangles) and MCF10A (green circles); (**d**) Loading plot of PC1.

We investigated the SERS spectra from the captured living MCF7 and MCF10A cells, as shown in Figure 5, using the AgNPs-rGO1-E4 SERS substrate for distinguishing the difference of the breast malignant cell (MCF7) and non-malignant cell (MCF10A). Briefly, we measured the Raman spectra of the MCF7 and MCF10A cells at 25 points within and nearby the cell nuclei followed by smoothing, background subtraction and normalization, denoted by light red and green lines in Figure 5a,b, respectively. The dark red and green lines represent the average Raman spectrum of each cells. In Figure 5a, the inset is an optical microscope (OM) image of the captured MCF7 cell that shows that the actin filaments were elongated by the enhanced surface structural interactions between the nano-undulated substrate and the cell components. This was not observed in the OM image of the MCF10A cell owing to the low EpCAM expression, as shown in Figure 5b. The Raman spectra of MCF7 and MCF10A cells on a glass substrate are depicted in Figure S9, which indicated that the distinct Raman band was not observed. Meanwhile, we obtained the enhanced distinguishable Raman peaks on the SERS substrate and the molecular assignment of Raman bands of the MCF7 and MCF10A cells and these are summarized in Table S2. The molecular assignment of the averaged spectra provided sufficient information about total cellular components [57,58]. In Figure 5a,b, Raman bands of proteins, lipids, nucleic acids and other carbohydrates are represented in red, green, blue and purple, respectively. Overall, Raman bands related to amino acids tended to be observed in longer wavelength ranges due to an increase in the percentage of the total Raman-active components in malignant cells [59,60]. The MCF7 cells in Figure 5a represented Raman peaks at 1237 and 1656 cm^−1^, corresponding to an amid I and amide III, which indicated that there were relatively high expressions of proteins inducing mitogenic activity in malignant cells compared with non-malignant cells [60,61]. The MCF7 cells also showed Raman bands of phenylalanine at 1003 and 1607 cm^−1^ indicating that there were more amino acid contents in malignant cells [62]. These results might be attributed to overexpressed cell membrane receptors such as transferrin receptors or folate receptors containing abundant phenylalanine, α-helix and β-sheet protein structures for maintaining the rapid cell growth and division of the malignant cells. On the other hand, we observed relatively few amino acid related Raman bands in the spectra of MCF10A cells, as shown in Figure 5b. We found the lipid related Raman bands at 1445 cm^−1^ for CH_2_CH_3_ bending modes of phospholipids where it was not observed in the spectra of MCF7 cells. Raman bands of 831 and 1173 cm^−1^ for tyrosine, 1043 cm^−1^ for proline and 1745 cm^−1^ for phospholipids in the MCF10A spectra also indicated the increased percentage of lipids in terms of the total Raman-active components in the non-malignant cells in comparison with the malignant cells [59,63].

To discriminate the spectral differences systemically, we carried out PCA on the spectra of the MCF7 and MCF10A cells and obtained similar features that were observed from the averaged spectra. Figure 5c represents the PCA score plot of PC1 and PC2. The score plot showed that the cancer cells were located on the positive side of the PC1 axis and the non-cancer cells located on the negative side, which meant that the transformed data onto the PC1 could distinguish the MCF7 and MCF10A cells. The loading plot of the PC1, as shown in Figure 5d, represented the components of the PC1 eigenvector; that is, positive and negative peaks of the loading plot were interrelated with the dots in the score plot locating on the positive or negative side of the PC1 axis, respectively. The positive peaks on the PC1 were at 621, 782, 842, 898, 950, 970, 1003, 1173, 1237, 1330, 1413, 1437, 1645 and 1737 cm^−1^, while the negative peaks on the PC1 were at 729, 827, 922, 1048, 1057, 1203, 1299, 1369, 1451, 1525, 1558, 1578, 1652 and 1660 cm^−1^. The positive or negative peaks were assigned by a previous study about Raman spectroscopy of biological tissues and indicated which cellular constituents significantly contributed to the Raman signals [58]. In Figure 5d, the Raman peaks in red, green, blue and purple describe the Raman signals attributed by proteins, lipids, nucleic acids and other carbohydrates, respectively. We observed that most of the positive Raman peaks arose from proteins (621, 950, 970, 1003, 1237, 1413 and 1646 cm^−1^) and most of the negative peaks arose from lipids (1057, 1294, 1369, 1451, 1652 and 1660 cm^−1^), which were similar features identified in the averaged spectra of MCF7 and MCF10A. According to our analysis, the positive values of PC1 showed spectral characteristics arising from proteins and Raman spectra of lipids affected the negative values of PC1 in the score plot. These results implied latent differences between cancerous and non-cancerous cells based on the Raman spectra corresponding to their protein and lipid components. It can be also surmised that the results are contributed by less mitotic activity of the non-cancer cells. Taken together, we demonstrated the SERS measurement of the captured MCF7 and MCF10A cells and successfully distinguished their biomolecular components without any post processes after capturing the cells. The developed AgNPs-rGO composite substrates may be utilized for analyzing other tumor-derived biomarkers such as DNAs, RNAs and exosomes as well as tumor cells with a further study of separation techniques and statistical methods [64].

## 4. Conclusions

We prepared the AgNPs-rGO SERS substrates using the simple, controllable and low-cost in situ electric field-assisted laser fabrication for efficient CTC capture and analysis without any post processes. Pulsed laser irradiation thermally reduced the mixture of AgNW and GO films into the AgNPs-rGO composites and formulated the 3-D nanostructured topography of the surface under the electric field-assisted environment. The synergistic effect of the biointerfacial properties of the rGO nanosheets and the nano-undulated surface of AgNPs-rGO films effectively mimicked the cellular microenvironment properties resulting in the high capture efficiency of CTCs. In addition, narrow nanogaps between the AgNPs greatly enhanced the Raman signals, which provided a platform to analyze the captured living cells for distinguishing cancer and non-cancer cells by SERS spectroscopy. Consequently, the suggested bio-inspired nano-undulated AgNPs-rGO composites showed bifunctional roles as an efficient cell capture biointerface and sensitive SERS substrate and will be applicable to cancer diagnosis or cell-based research in future with further study of other tumor-derived biomarkers.

## Figures and Tables

**Table 1:** Roughness of the fabricated AgNPs-rGO composites using different DC electric fields.

Composite	Applied Voltage (V)	Electric Field (V/m)	Roughness (nm)
AgNPs-rGO1	0	0	19 ± 3
AgNPs-rGO1-E1	7.5	2500	436 ± 31
AgNPs-rGO1-E2	15	5000	552 ± 49
AgNPs-rGO1-E3	22.5	7500	689 ± 54
AgNPs-rGO1-E4	30	10,000	521 ± 36

## Supplementary Materials

The following are available online at https://www.mdpi.com/1424-8220/20/18/5089/s1, Figure S1: Fluorescence microscope images of the captured MCF7 cells, Figure S2: Raman spectra of the malignant MCF7 cells and non-malignant MCF10A cells on microscope slides, Figure S3: (a,b) Raman spectra of R6G solutions with different concentrations on the AgNPs-rGO1 SERS substrates; (c) Intensity plot of the 612 cm^−1^ as a function of R6G concentration in logarithmic scale. Red line is the linear regression equation, Figure S4: (a,b) Raman spectra of R6G solutions with different concentrations on the AgNPs-rGO1 SERS substrates; (c) Intensity plot of the 612 cm^−1^ as a function of R6G concentration in logarithmic scale. Red line is the linear regression equation, Figure S5: Stability test of the AgNPs-rGO1 SERS substrate. (a) The SERS spectra of 1 μM R6G solutions during a month; (b–e) The SERS intensity of R6G at the 612 cm^−1^, 773 cm^−1^, 1362 cm^−1^, and 1650 cm^−1^ over time, Figure S6: Alexa Fluor 488 dye conjugated antibodies on the substrates. The anti-EpCAM antibodies was functionalized on the substrate through avidin-biotin interaction. The functionalization layer with thickness of ~10 nm involving the uniformly distributed antibodies adhere to the cell surface of 3-D structure resulting in the Raman enhancement of cell components near the substrates, Figure S7: Alexa Fluor 488 dye conjugated antibodies on the substrates. The anti-EpCAM antibodies was functionalized on the substrate through avidin-biotin interaction. The functionalization layer with thickness of ~10 nm involving the uniformly distributed antibodies adhere to the cell surface of 3-D structure resulting in the Raman enhancement of cell components near the substrates, Figure S8: (a) Contact angle of the AgNWs film and the AgNWs-GO1 film. Inset photographs are water droplets on the films; (b) Stiffness of the AgNWs film and the AgNWs-GO1 film. The films was tested in indent depth of 100 nm, Figure S9: (a) Contact angle of the AgNWs film and the AgNWs-GO1 film. Inset photographs are water droplets on the films; (b) Stiffness of the AgNWs film and the AgNWs-GO1 film. The films was tested in indent depth of 100 nm, Table S1: Assignment of Raman bands of the MCF7 and MCF10A cells, Table S2: Molecular assignment of Raman bands of the MCF7 and MCF10A cells.
