# Supplementary material for: Efficient Capture and Raman Analysis of Circulating Tumor Cells by Nano-Undulated AgNPs-rGO Composite SERS Substrates
Source: Sensors (Basel). 2020 Sep 7;20(18):5089. doi: 10.3390/s20185089 (PMC7570931; doi:10.3390/s20185089)
Supplement: Supplementary file 1 [file sensors-20-05089-s001.pdf]

sensors

MDPI

Supplementary Materials

Efficient Capture and Raman Analysis of Circulating Tumor Cells by Nano-Undulated AgNPs-rGO Composite SERS Substrates

Jong-Eun Park [1,†], Nuri Oh [2,3,†], Hyeono Nam [1], Ji-Ho Park [2], Sanha Kim [1], Jessie S. Jeon [1,*] and Minyang Yang [1,4,*]

[1] Department of Mechanical Engineering, Korea Advanced Institute of Science and Technology, Daejeon 34141, Republic of Korea; pje7031@kaist.ac.kr (J.-E.P.); namtree@kaist.ac.kr (H.N.); sanhkim@kaist.ac.kr (S.K.); jsjeon@kaist.ac.kr (J.S.J.); myyang@kaist.ac.kr (M.Y.)

[2] Department of Bio and Brain Engineering, Korea Advanced Institute of Science and Technology, Daejeon 34141, Republic of Korea; ohnuri@kaist.ac.kr (N.O.); jihopark@kaist.ac.kr (J.-H.P.)

[3] Center for Systems Biology, Massachusetts General Hospital, Boston, Massachusetts 02114, United States; ohnuri@kaist.ac.kr (N.O.)

[4] Department of Mechanical Engineering, State University of New York Korea, Incheon 21985, Republic of Korea; minyang.yang@sunykorea.ac.kr (M.Y.)

* Correspondence:jsjeon@kaist.ac.kr (J.S.J.); myyang@kaist.ac.kr (M.Y.)

† These authors contributed equally to this work.

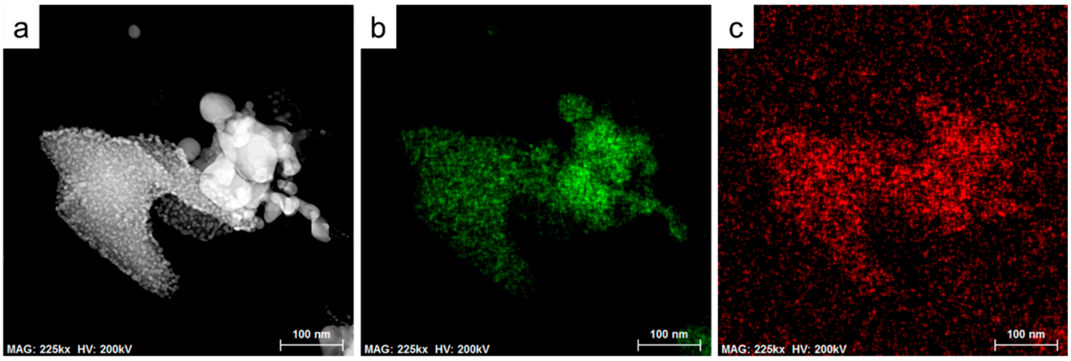

Figure S1. (a) HAADF-STEM image of the AgNPs-rGO composite; Corresponding elemental maps of silver **(b)**; carbon **(c)**.

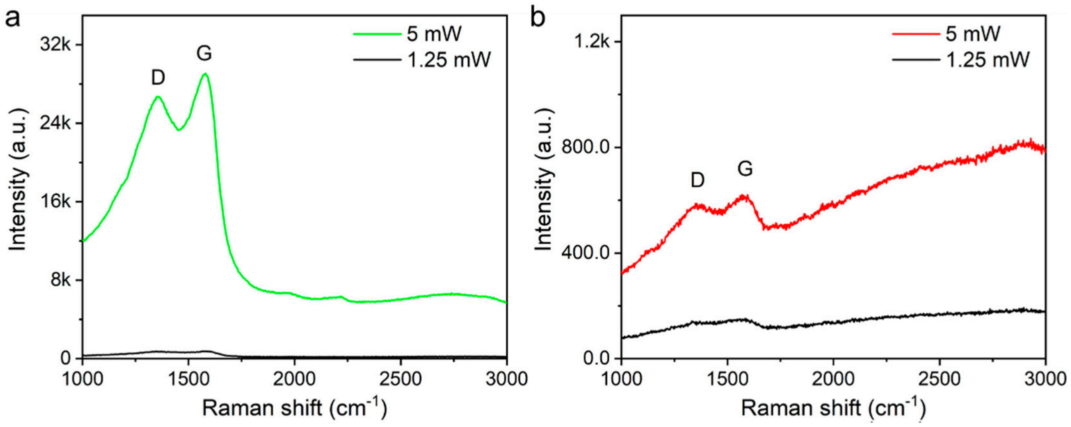

Figure S2. Raman spectra of the AgNPs-rGO1 substrate with high and low input laser power at wavelength of 514 nm **(a)**; 633 nm **(b)**. The raw data was shown for the background verification.

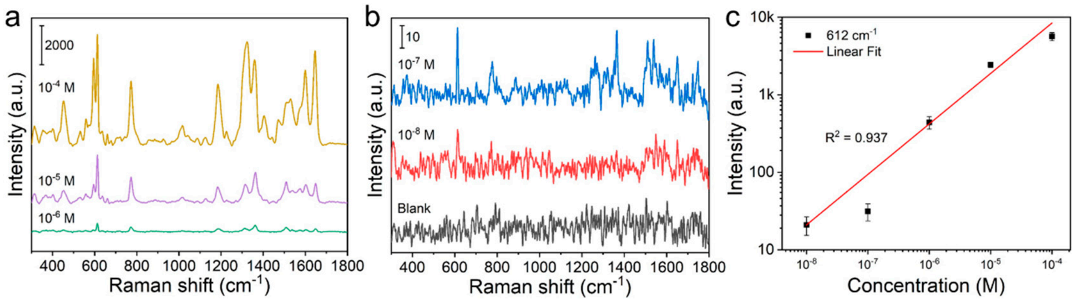

Figure S3. (a,b) Raman spectra of R6G solutions with different concentrations on the AgNPs-rGO1 SERS substrates; **(c)** Intensity plot of the 612 cm^{-1} as a function of R6G concentration in logarithmic scale. Red line is the linear regression equation.

For the enhancement factor (EF) calculation, I_{SERS} = 20.97, I_{bulk} = 580.27 at 612 cm^{-1}.

N_{bulk} was calculated as follows [1],

$$N_{bulk} = Ah\rho/M \quad (1)$$

where A is the laser spot area (1.81 μm²), h is the laser penetration depth (22.9 μm for 633 nm), ρ and M are the density (1.26 g/cm³) and molecular weight (479.02 g/mol) of R6G, respectively. Thus, N_{bulk} = 1.09 × 10^{-13} mol.

N_{sers} was calculated as below [1],

$$N_{SERS} = CVA/S \quad (2)$$

where C is the R6G concentration (10 nM), V is the sample volume (1 μL), A is the laser spot area (1.81 μm²), and is the sample area (2.24 mm²). Thus, N_{SERS} = 8.08 × 10^{-21} mol.

Based on the calculation, EF = (20.97/580.27) × (1.09 × 10^{-13})/(8.08 × 10^{-21}) = 4.88 × 10^5.

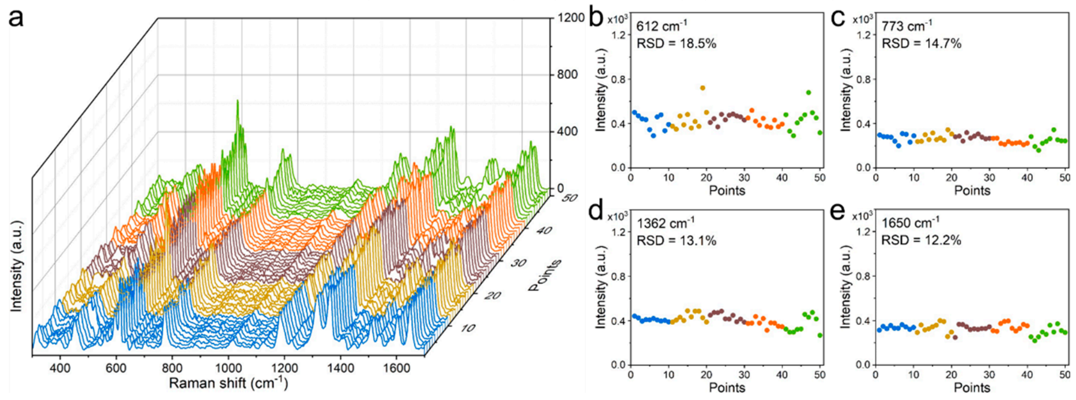

Figure S4. Reproducibility of the AgNPs-rGO1 SERS substrate. **(a)** SERS spectra of R6G from randomly selected 50 points. Five samples were measured with 10 points per the each sample; SERS intensity distribution of the R6G at the Raman peaks of 612 cm^{-1} **(b)**; 773 cm^{-1} **(c)**; 1362 cm^{-1} **(d)**; 1650 cm^{-1} **(e)**. Different colors represent the five each sample.

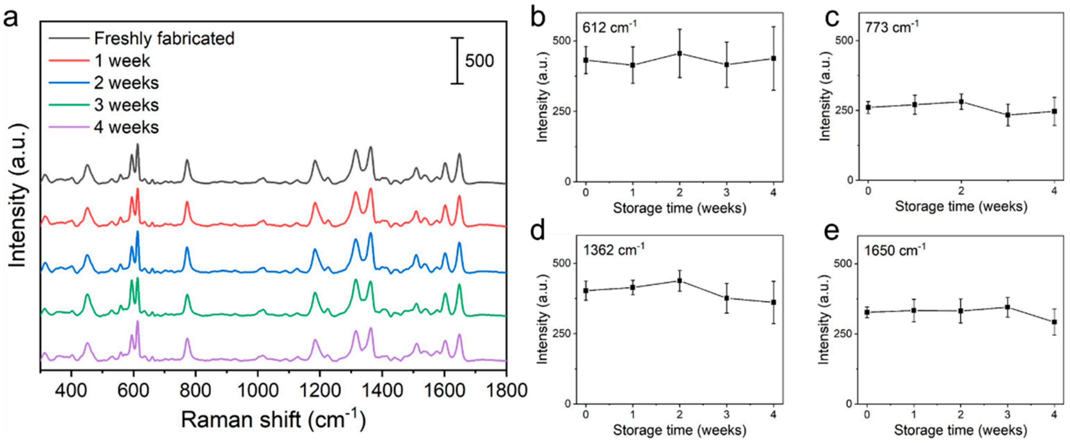

Figure S5. Stability test of the AgNPs-rGO1 SERS substrate. **(a)** The SERS spectra of 1 μM R6G solutions during a month; **(b–e)** The SERS intensity of R6G at the 612 cm^{-1}, 773 cm^{-1}, 1362 cm^{-1}, and 1650 cm^{-1} over time.

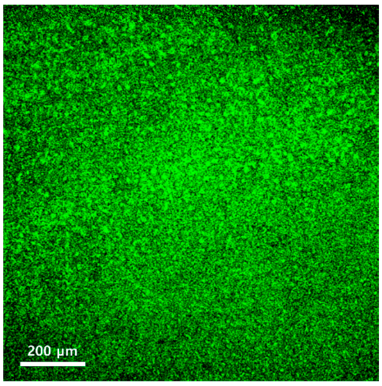

Figure S6. Alexa Fluor 488 dye conjugated antibodies on the substrates. The anti-EpCAM antibodies was functionalized on the substrate through avidin-biotin interaction. The functionalization layer with thickness of ~10 nm involving the uniformly distributed antibodies adhere to the cell surface of 3-D structure resulting in the Raman enhancement of cell components near the substrates.

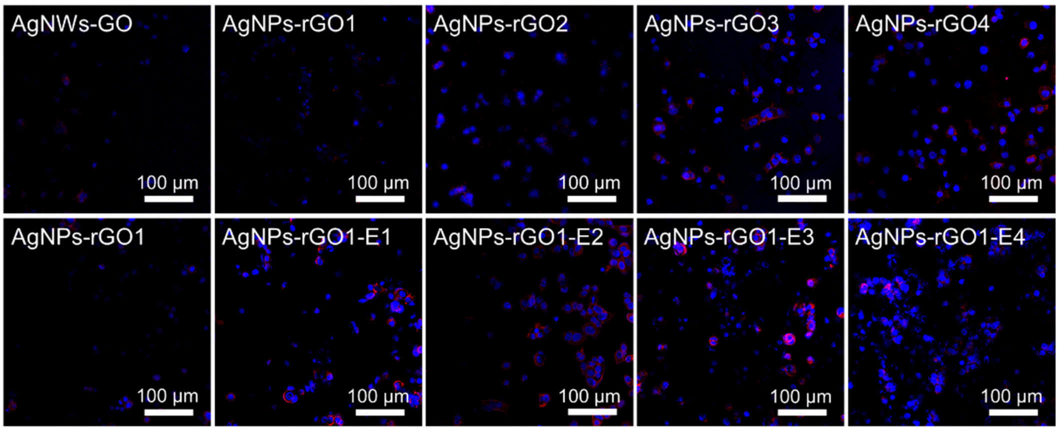

Figure S7. Fluorescence microscope images of the MCF7 cells stained with DAPI (blue) for nuclei and rhodamine-phalloidin (red) for actin filaments on the various SERS substrates after 1 hour capture time. The SERS substrates were fabricated using the AgNWs-GO mixture films with the different GO contents (top) and different applied electric fields during the process (bottom).

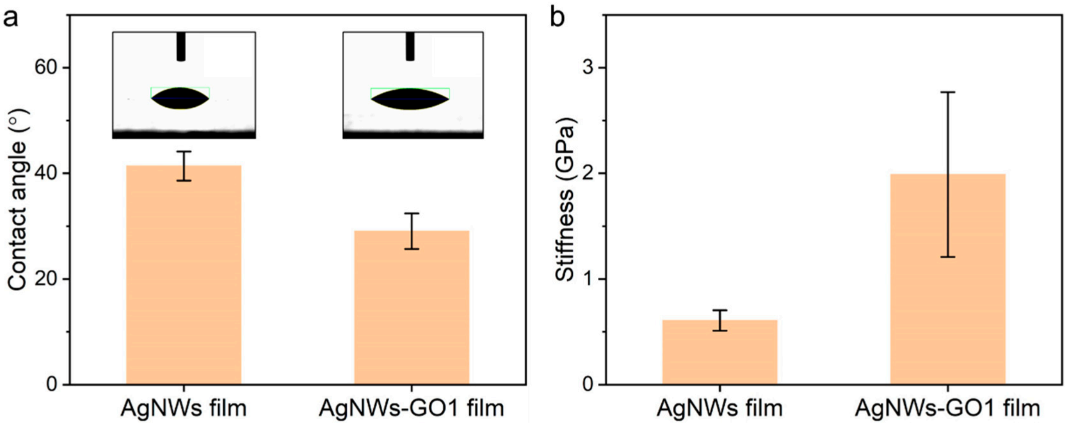

Figure S8. (a) Contact angle of the AgNWs film and the AgNWs-GO1 film. Inset photographs are water droplets on the films; **(b)** Stiffness of the AgNWs film and the AgNWs-GO1 film. The films was tested in indent depth of 100 nm.

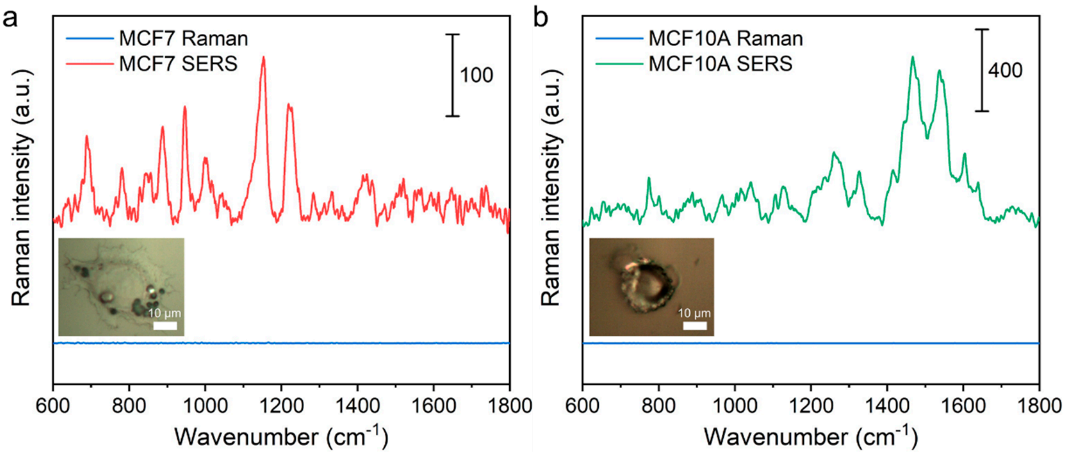

Figure S9. Comparison of the enhanced and non-enhanced Raman spectra. **(a)** Raman spectrum of a MCF7 cell on the AgNPs-rGO1 SERS substrate (red line) and on a microscope slide (blue line); **(b)** Raman spectrum of a MCF10A cell on the AgNPs-rGO1 SERS substrate (green line) and on a microscope slide (blue line). Inset is the MCF7 and MCF10A cell on the microscope slides.

Table S1. Comparison of LOD of R6G and the other methods.

	**Liquid Chromatography**	**Solid-phase Extraction**	**Fluorescence Sensor**	**Other SERS Method**	**This Work**
LOD	2.7 nM [2]	2.1 μg/L [3] (4.4 nM)	0.26 μM [4]	0.1 nM [5]	2.0 nM

Table S2. Molecular assignment of Raman bands of the MCF7 and MCF10A cells.

Raman Shift (cm^{-1})	Molecular Assignment	Cell Type	References
621	C–C twisting mode of phenylalanine (proteins)	MCF7	6,7,8
630	Glycerol	MCF10A	9
662	C–S stretching mode of cystine (collagen type I)	MCF10A	6,10
702	Cholesterol, cholesterol ester	MCF7	9
709	ν(C–S) trans (amino acid methionine)	MCF10A	11
782	Thymine, cytosine, uracil	MCF7	8
802	Uracil-based ring breathing mode	MCF10A	12
831	Asymmetric O–P–O stretching, tyrosine	MCF10A	13
842	Glucose	MCF7	7
877	C–C–N^+ symmetric stretching (lipids)	MCF10A	8
883	$\rho(CH_2)$ (protein assignment)	MCF7	14
893	Backbone, C–C skeletal	MCF10A	13
957	Hydroxyapatite, carotenoid, cholesterol	MCF10A	7
970	Phosphate monoester groups of phosphorylated proteins and cellular nucleic acids	MCF7	15
980	C–C stretching β-sheet (proteins)	MCF10A	8
1003	Phenylalanine, C–C skeletal	MCF7	13
1007	Phenylalanine, carbamide	MCF10A	16
1040	Formalin peaks appearing in the fixed normal and tumor tissues	MCF7	17
1043	Proline (collagen assignment)	MCF10A	18,19
1131	Fatty acid	MCF7, MCF10A	9
1155	C–C (and C–N) stretching of proteins (also carotenoids)	MCF7	6,7
1173	Tyrosine (collagen type I)	MCF10A	9
1237	Amide III and CH_2 wagging vibrations from glycine backbone and proline side chains	MCF7	15
1330	Typical phospholipids	MCF7	20
1350	Carbon particle	MCF10A	21
1359	Tryptophan	MCF10A	10
1409	ν_s COO^-	MCF10A	22
1413	ν(C=O)O^- (amino acids, aspartic and glutamic acid)	MCF7	11
1445	CH_2CH_3 bending modes of collagen and phospholipids	MCF10A	23,24
1472	Paraffin	MCF7	15
1525	In-plane vibrations of the conjugated −C=C−	MCF10A	25
1528	Carotenoid (absent in normal tissues)	MCF7	26
1554	Amide II	MCF10A	27
1573	Guanine, adenine, TRP (protein)	MCF7	7
1585	C=C olefinic stretch (protein assignment)	MCF7	28
1602	Phenylalanine	MCF10A	23
1607	Tyrosine, phenylalanine ring vibration	MCF7	22
1653	Lipid (C=C stretch)	MCF10A	7
1656	Amide I (proteins)	MCF7	22
1725	C=O lipids	MCF10A	29
1738	Lipids	MCF7	15
1745	Lipids	MCF10A	30

References

1. Rao, V. K.; Radhakrishnan, T. P. Tuning the SERS response with Ag-Au nanoparticle-embedded polymer thin film substrates. *Appl. Mater. Interfaces* **2015**, *7*, 12767–12773.
2. Xie, J.; Xie, J.; Deng, J.; Fang, X.; Zhao, H.; Qian, D.; Wang, H. Computational design and fabrication of core–shell magnetic molecularly imprinted polymer for dispersive micro-solid-phase extraction coupled with high-performance liquid chromatography for the determination of rhodamine 6G. *J. Sep. Sci.* **2016**, *39*, 2422–2430.
3. Chao, Y.; Pang, J.; Yan, B.; Wu, P.; Luo, J.; He, J.; Jin, Y.; Li, X.; Xiong, J.; Li, H.; Zhu, W. Graphene-like BN@SiO_2 nanocomposites as efficient sorbents for solid-phase extraction of Rhodamine B and Rhodamine 6G from food samples. *Food Chem.* **2020**, *320*, 126666.
4. Li, Y.; He, W.; Peng, Q.; Hou, L.; He, J.; Li, K. Aggregation-induced emission luminogen based molecularly imprinted ratiometric fluorescence sensor for the detection of Rhodamine 6G in food samples. *Food Chem.* **2019**, *287*, 55–60.
5. Fan, W.; Lee, Y. H.; Pedireddy, S.; Zhang, Q.; Liu, T.; Ling, X. Y. Graphene oxide and shape-controlled silver nanoparticle hybrids for ultrasensitive single-particle surface-enhanced Raman scattering (SERS) sensing. *Nanoscale* **2014**, *6*, 4843–4851.
6. Stone, N.; Kendall, C.; Shepherd, N.; Crow, P.; Barr, H. Near-infrared Raman spectroscopy for the classification of epithelial pre-cancers and cancers. *J. Raman Spectrosc.* **2002**, *33*, 564–573.
7. Stone, N.; Kendall, C.; Smith, J.; Crow, P.; Barr, H. Raman spectroscopy for identification of epithelial cancers. *Faraday Discuss.* **2004**, *126*, 141–157.
8. Liu, Z.; Davis, C.; Cai, W.; He, L.; Chen, X.; Dai, H. Circulation and long-term fate of functionalized, biocompatible single-walled carbon nanotubes in mice probed by Raman spectroscopy. *Proc. Natl. Acad. Sci. Unit. States Am.* **2008**, *105*, 1410–1415.
9. Krafft, C.; Neudert, L.; Simat, T.; Salzer, R. Near infrared Raman spectra of human brain lipids. Spectrochim. *Acta Mol. Biomol. Spectrosc.* **2005**, *61*, 1529–1535.
10. Cheng, W. T.; Liu, M. T.; Liu, H. N.; Lin, S. Y. Micro-Raman spectroscopy used to identify and grade human skin pilomatrixoma. *Microsc. Res. Tech.* **2005**, *68*, 75–79.
11. Kendall, C.; Day, J.; Hutchings, J.; Smith, B.; Shepherd, N.; Barr, H.; Stone, N. Evaluation of Raman probe for oesophageal cancer diagnostics. *Analyst* **2010**, *135*, 3038–3041.
12. Chan, J. W.; Taylor, D. S.; Zwerdling, T.; Lane, S. M.; Ihara, K.; Huser, T. Micro-Raman spectroscopy detects individual neoplastic and normal hematopoietic cells. *Biophys. J.* **2006**, *90*, 648–656.
13. Bhattacharjee, T.; Kumar, P.; Maru, G.; Ingle, A.; Krishna, C. M. Swiss bare mice: A suitable model for transcutaneous in vivo Raman spectroscopic studies of breast cancer. *Laser. Med. Sci.* **2014**, *29*, 325–333.
14. Dukor, R. K. Vibrational spectroscopy in the detection of cancer. *Handb. of Vib. Spectrosc.* **2001**.
15. Li, X.; Yang, T.; Li, S.; Yu, T. Surface-enhanced Raman spectroscopy differences of saliva between lung cancer patients and normal people. *Conf. on Clinical and Biomedical Spectroscopy and Imaging II* **2011**, Munich, Germany, May 22.
16. Notingher, I.; Green, C.; Dyer, C.; Perkins, E.; Hopkins, N.; Lindsay, C.; Hench, L. L. Discrimination between ricin and sulphur mustard toxicity in vitro using Raman spectroscopy. *J. R. Soc. Interface* **2004**, *1*, 79–90.
17. Bonnier, F.; Byrne, H. J. Understanding the molecular information contained in principal component analysis of vibrational spectra of biological systems. *Anal.* **2012**, *137*, 2.
18. Frank, C. J.; McCreery, R. L.; Redd, D. C. B. Raman-spectroscopy of normal and diseased human breast tissues. *Anal. Chem.* **1995**, *67*, 777–783.
19. Malini, R.; Venkatakrishna, K.; Kurien, J.; Pai, K. M.; Rao, L.; Kartha, V. B.; Krishna, C. M. Discrimination of normal, inflammatory, premalignant, and malignant oral tissue: A Raman spectroscopy study. *Biopolym.* **2006**, *81*, 179–193.
20. Min, Y. K.; Yamamoto, T.; Kohda, E.; Ito, T.; Hamaguchi, H. 1064 nm near-infrared multichannel Raman spectroscopy of fresh human lung tissues. *J. Raman Spectrosc.* **2005**, *36*, 73–76.
21. Lakshmi, R. J.; Kartha, V. B.; Krishna, C. M.; Solomon, J. G. R.; Ullas, G.; Devi, P. U. Tissue Raman spectroscopy for the study of radiation damage: Brain irradiation of mice. *Radiat. Res.* **2002**, *157*, 175–182.
22. Shetty, G.; Kendall, C.; Shepherd, N.; Stone, N.; Barr, H. Raman spectroscopy: Elucidation of biochemical changes in carcinogenesis of oesophagus. *Br. J. Cancer* **2006**, *94*, 1460–1464.

23. Huang, Z. W.; McWilliams, A.; Lui, H.; McLean, D. I.; Lam, S.; Zeng, H. S. Near-infrared Raman spectroscopy for optical diagnosis of lung cancer. *Int. J. Cancer* **2003**, *107*, 1047–1052.
24. Feng, S.; Pan, J.; Wu, Y.; Lin, D.; Chen, Y.; Xi, G.; Lin, J.; Chen, R. Study on gastric cancer blood plasma based on surface-enhanced Raman spectroscopy combined with multivariate analysis. *Sci. China C Life Sci.* **2011**, *54*, 828–834.
25. Puppels, G. J.; Garritsen, H. S. P.; Kummer, J. A.; Greve, J. Carotenoids located in human lymphocyte subpopulations and natural-killer-cells by Raman microspectroscopy. *Cytom.* **1993**, *14*, 251–256.
26. Mahadevan-Jansen, A.; Richards-Kortum, R. Raman spectroscopy for cancer detection: A review. *International Conference of the IEEE Engineering in Medicine and Biology Society* **1997**, Chicago, IL, October 30–November 2.
27. O Faolain, E.; Hunter, M. B.; Byrne, J. M.; Kelehan, P.; McNamara, M.; Byrne, H. J.; Lyng, F. M. A study examining the effects of tissue processing on human tissue sections using vibrational spectroscopy. *Vib. Spectrosc.* **2005**, *38*, 121–127.
28. Sahu, A.; Dalal, K.; Naglot, S.; Aggarwal, P.; Krishna, C. M. Serum based diagnosis of asthma using Raman spectroscopy: An early phase pilot study. *PLos ONE* **2013**, *8*, e78921.
29. Huang, N. Y.; Short, M.; Zhao, J. H.; Wang, H. Q.; Lui, H.; Korbelik, M.; Zeng, H. S. Full range characterization of the Raman spectra of organs in a murine model. *Opt. Express* **2011**, *19*, 22892–22909.
30. Ronen, S. M.; Stier, A.; Degani, H. NMR-studies of the lipid-metabolism of T47d human breast-cancer spheroids. *FEBS Lett.* **1990**, *266*, 147–149.
